# A view not to be missed: Salient scene content interferes with cognitive restoration

**DOI:** 10.1371/journal.pone.0169997

**Published:** 2017-07-19

**Authors:** Alexander P. N. Van der Jagt, Tony Craig, Mark J. Brewer, David G. Pearson

**Affiliations:** 1 University of Aberdeen, Aberdeen, United Kingdom; 2 The James Hutton Institute, Aberdeen, United Kingdom; 3 Biomathematics and Statistics Scotland, Aberdeen, United Kingdom; 4 Anglia Ruskin University, Cambridge, United Kingdom; University of Akron, UNITED STATES

## Abstract

Attention Restoration Theory (ART) states that built scenes place greater load on attentional resources than natural scenes. This is explained in terms of "hard" and "soft" fascination of built and natural scenes. Given a lack of direct empirical evidence for this assumption we propose that perceptual saliency of scene content can function as an empirically derived indicator of fascination. Saliency levels were established by measuring speed of scene category detection using a Go/No-Go detection paradigm. Experiment 1 shows that built scenes are more salient than natural scenes. Experiment 2 replicates these findings using greyscale images, ruling out a colour-based response strategy, and additionally shows that built objects in natural scenes affect saliency to a greater extent than the reverse. Experiment 3 demonstrates that the saliency of scene content is directly linked to cognitive restoration using an established restoration paradigm. Overall, these findings demonstrate an important link between the saliency of scene content and related cognitive restoration.

## Introduction

There is widespread empirical support for the notion that exposure to natural environments is cognitively restorative: aiding “the renewal or recovery of resources or capacities that have become depleted in meeting the demands of everyday life” ([1], p. 42). For instance, a nature visit has been shown to improve executive task performance more strongly than a visit to a built environment [2–4]. This finding has been replicated using video and image presentations of natural and built environments [5–7], suggesting that cognitive restoration does not necessarily require a multi-sensory environmental experience.

To account for the restorative potential of natural environments, common reference is made to attention restoration theory (ART; R. [8–10]. This theory claims that the built environment invokes stronger bottom-up attentional capture (i.e. *hard fascination*) than the natural environment due to a ubiquity of irrelevant, yet fascinating, stimuli. This is purported to lead to the expenditure of executive attention resources on top-down control of behaviour [5, 10]. Despite its popularity, there is no direct empirical support for the central idea in ART that environments eliciting strong attentional capture interfere with restoration from mental fatigue.

Here we propose that *scene saliency* [11–12] can function as an empirically-derived indicator of the degree to which (elements within) environments invoke hard or soft fascination; attentional capture is considered to be a function of stimulus saliency ([13], but see [14], for an alternative view). Although not typically used towards this end, we believe for reasons outlined below that saliency levels can be established by measuring speed of scene category detection using a Go/No-Go detection paradigm (e.g. [15–16]).

### Speeded scene detection as an indicator of saliency

The Go/No-Go detection paradigm involves participants detecting briefly flashed images from a pre-specified target category as quickly as possible while ignoring distractor images. This type of task is typically deployed to study the processing time of a scene's global lay-out properties and category (e.g., a beach or a forest), which is referred to as the scene gist [17]. This initial, abstract scene representation may also include elementary features such as luminance, edges (i.e., orientation), contrast and chromacity (i.e., colour) that are of a salient nature [11–18]. Importantly, research suggests that such elementary features might be represented to different degrees in natural and built environments. Typical modern, Western, built environments are dominated by straight contours and smooth surfaces, whereas natural environments are characterized by undulating, smooth contours and textured surfaces [19–20]. Contrast levels are also different with more "sharp discontinuities and abrupt transitions" ([21], p. 15) in built scenes as they tend to lack the strong correlation in luminance value, as well as the orientation, of neighbouring pixels typical of natural scenes [22–23]. If ART is correct, we would predict low-level features of built environments to be more salient than low-level features of natural environments. A brief exposure time is sufficient to generate awareness and rudimentary identification of objects with such salient features. For example, exposure to an image showing an interior of a house for only 27 ms followed by a backward mask was sufficient for participants to report "square things, maybe furniture" in a free recall task [24]. Research has also demonstrated that people use such local information, along with global information (i.e. the distribution of low-level features such as orientation, colour or texture throughout the whole scene), in speeded scene and object detection tasks [25–27]. For example, an image manipulation which distorts low-level scene features reduced the speed at which scenes could be detected as either natural or built [26].

A potential limitation of the Go/No-Go detection paradigm is that factors other than saliency could influence speed of detection. This might be elements (e.g., fearful stimuli) for which we have evolved specialized detection modules [28] or that are familiar due to prior experience in an experimental setting [29–30] or in real life [31]. Images also vary in low-level features that could inadvertently influence detection performance [11]. For example, a recent study showed a relationship between mean contrast energy in natural and built scenes and response time following brief exposures, with scenes high in contrast energy more likely to be built scenes [32]. In the present study, these concerns have been addressed by selecting a very substantial and variable set of natural and built images depicting local, familiar settings. This approach was preferred over controlling images for low-level features because it allowed saliency to be approximated as it naturally varies between natural and built scene content. In addition, we also statistically controlled for participants' experience living in rural or urban environments, which could influence familiarity with either natural or built scenes.

### The present study

The first aim of the study was to investigate if built scenes indeed have more diagnostic salient features than natural scenes, which could potentially be important to predicting the restorative qualities of a scene by triggering attentional capture, using the Go/No-Go detection paradigm. As many of our everyday environments comprise a mixture of natural and built content, it was also explored how semantically *inconsistent* elements (i.e. built elements within a natural scene or *vice versa*—electricity pylons within a field; trees within a street) impact on speeded scene detection. Inconsistent scenes can be contrasted with consistent scenes in which there are no inconsistent elements. The extent to which an inconsistent object interferes with the speed of scene category detection is likely to be higher for objects from the built—more salient–category [15]. A second aim of this study, and a direct test of ART, was to study if salient elements in a scene negatively impact on restoration from mental fatigue.

Go/No-Go studies have previously been undertaken to scrutinize whether evolutionary relevant animal stimuli are detected more quickly than artificial ‘means of transport’ stimuli [33]. Other studies employed the Go/No-Go paradigm in combination with natural and built scene presentations (with and without semantically inconsistent objects; [15–16, 26]. However, these studies were not specifically designed to contrast peak saliency levels in natural and built scenes, and the findings are inconclusive in terms of the predictions made by ART. The present research therefore took an alternative approach to image selection by classifying the scene category of images, setting exposure times and applying backward masking. For example, we avoided commercial image libraries, instead selecting images of everyday mostly mundane scenes from different online resources. In addition, we checked for inter-rater agreement on scene labels, which showed that people had stronger agreement on the meaning of the concepts 'natural' and 'built' than alternative concepts such as 'pristine' or man-made'. Hence, we chose 'natural' and 'built' as the response categories in the Go/No-Go task. A second pilot study served to allocate images to validate the four image categories: *natural consistent; natural inconsistent*, *built consistent and built inconsistent* by an independent panel. We discarded any images with low high inter-rater agreement or borderline classifications of natural and built scene content. A full description of how the approach to image selection and classifying scene category diverged from previous practice in Go/No-Go studies is provided in the online Supporting Information.

Previous natural/built Go/No-Go studies have typically presented natural and built images at very brief exposure times within the 20–27 ms range with no backward masking [15–16, 26]. However, this practice could have enabled ongoing processing of scene information after image offset due to the storage of visual information in a sensory buffer (i.e. iconic memory; [34]). This ongoing processing of an “afterimage” can be interrupted by presenting a new visual stimulus (i.e. a backward mask) within a short time interval of the experimental stimulus. Backward masking is thus of relevance when investigating what stimulus content is most salient. Therefore, the image presentation procedure of the present study included backward masking. A previous study using speeded detection tasks including backward masking demonstrated that the natural and built scene category of images could be classified with 75% accuracy after just 19 ms of exposure time [35]). Another study showed above chance performance in a natural/man-made categorization after a 12 ms exposure followed by a mask with a 12 ms stimulus onset asynchrony [36], while a third study showed that masked image presentations of 27 ms suffice to discriminate natural from built scenes above chance level [37]. Since it has not yet been established what exposure time threshold is required for stimulus saliency to affect natural/built scene detection in a Go/No-Go task using a set of *consistent* and *inconsistent* scenes, images were presented at five different exposure times within the 13–67 ms range.

The first two experiments tested the hypothesis that scene detection (natural or built) is faster for built than natural scenes in a Go/No-Go task. In addition, we envisaged the detrimental effect of an object that is inconsistent with superordinate scene category to be larger in natural (with a built object) than in built (with a natural object) scenes. A third experiment was run to directly test the central idea in ART that non-salient scenes are more cognitively restorative than salient scenes, using images classified as having salient or non-salient features on the basis of response times in previous Go/No-Go experiments. This was done by investigating how exposure to slideshows with images of either high or low saliency influences performance on the Backward Digit Span task, which was employed as a measure of directed attention [5].

## Ethics statement

Written informed consent was obtained from all participants prior to data collection and the study received ethical approval from University of Aberdeen Psychology Ethics Panel, following the principles of the British Psychological Society and the Declaration of Helsinki.

## Experiment 1

### Method

#### Participants and design

Forty students (25 female) with a mean age of 21.7 years (*SE* = .59) were recruited from the University of Aberdeen. Participation was rewarded with course credit. All participants had normal or corrected-to-normal vision. Data collection ceased once the pre-determined sample size based on similar Go/No-Go experiments with natural and built images (e.g., [15]), taking into account the higher number of exposure times, was reached.

The experiment used a 2 (Target Category: Natural or Built) x 2 (Consistency: Consistent or Inconsistent) within-subjects design.

#### Materials

A total of 1600 full-colour images were selected from two online image repositories: SCRAN (Scottish Cultural Resources Access Network, http://www.scran.ac.uk) and Flickr Creative Commons (http://www.flickr.com); 400 representative of each of the four image categories (*consistent natural*, *consistent built*, *inconsistent natural*, *inconsistent built*). All images were resized to a resolution of 668 x 501 pixels (or 501 x 668 when vertically orientated). Scene category of images was validated using a pilot study (100 participants; 71 female; *M* = 20.6 years old; SE = .53). This resulted in a 1060-image (50% natural) database with both consistent and inconsistent images that had been reliably categorized as either natural or built. A detailed description of the image validation procedure is provided in S1 Supporting Information.

#### Procedure

**Fig 1:**
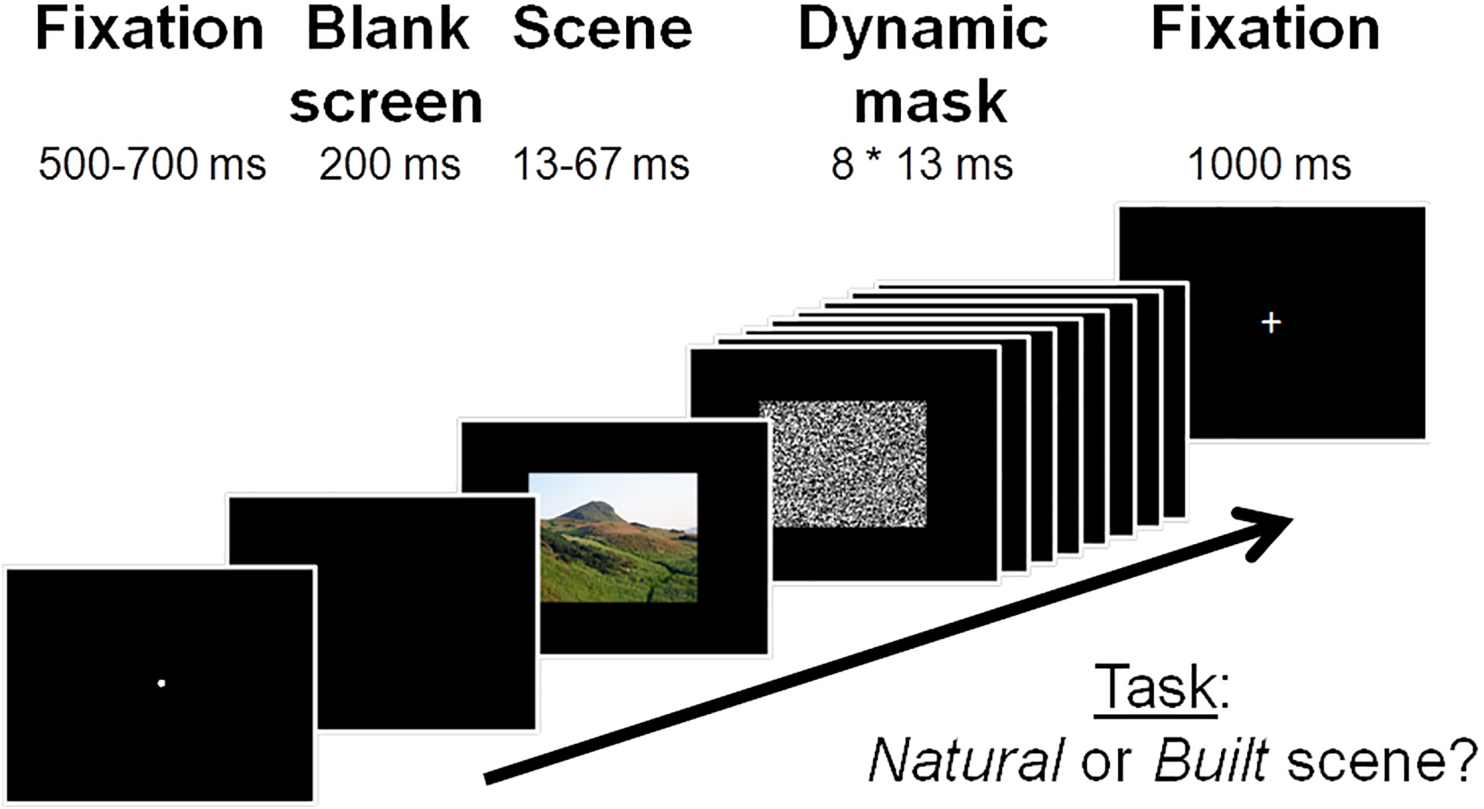
A schematic overview of a trial in the Go/No-Go task.

A Go/No-Go paradigm was employed with natural and built target and distracter images. Participants were instructed to push and hold a pre-assigned button on a keypad, upon which a fixation dot appeared, remaining on the screen for a duration randomly set between 500–700 ms. Following a brief blank screen (200 ms), either a natural or a built image was displayed for a duration of 13, 27, 40, 53 or 67 ms (randomly set). This image was replaced by a dynamic mask, consisting of eight randomly selected white noise images (from a set of 16), presented without inter-stimulus interval for a duration of 104 ms (8*13 ms). Finally, a fixation cross was displayed for 1,000 ms in order to provide participants with additional time to make a response (see Fig 1).

Participants were instructed to respond to target images (presented in 50% of all trials) by lifting their finger from the button as quickly as possible (“Go”-response) and to distractor images by continuing to hold the button pressed down (“No-Go”-response). Following a “Go”-response, a new trial could be initiated by pressing the button. A new trial was initiated automatically following a “No-Go”-response.

The experiment commenced with a 20-trial training block with exposure time set at 67 ms. Participants received immediate feedback on any errors made and the training block was looped until performance was above a threshold of 80 percent correct. Midway through the experiment, target category was switched and participants completed training once again before onset of the second block of trials. Within a block of trials an equal number of natural and built images were presented at each of the five exposure times. Each participant completed 1060 experimental trials. Hence, a group of 10 participants was required for an image to be presented once as a target and as a distractor at each of the exposure times (1060 images x 5 exposure times x 2 trial types = 10600 trials). In addition, 60 mask-only control trials were randomly interspersed amongst the experimental trials. These served to check if participants associated a lack of salient of information with natural scenes.

#### Data collection and analysis

The present research was conducted in brightly lit computer cubicles without daylight. The experiment was run on the software E-Prime 2.0 (Psychology Software Tools, Sharpsburg, PA). Trials were displayed on a 19-inch flat panel monitor (Dell Inc., Round Rock, TX). The screen resolution of the monitor was set at 1280 x 1024 pixels with a refresh rate of 75 Hz. All participants were seated at 100 cm from the computer screen. Images displayed on the screen subtended 10° x 7.5° (or 7.5° x 10° when vertically oriented) of visual angle. All statistical analyses (mixed models with fixed and random effects) have been run using the *glmer* program (*lme4* package; [38]) in the R system for statistical analysis [39].

Random effects capture and remove systematic variance above and beyond the effects of the included predictor variables in the regression equation. Random effects are ideally drawn upon when one wishes to generalize findings obtained with a specific sample used in an experiment to the wider population, when observations are nested within observers and when there are multiple observations from each participant [40]. Such generalization was also a requirement in the present study since each participant was presented with a different set of images at each of the exposure times. That is, more than one participant was required for each image to be presented at each of the exposure times for a single time. Random effects ruled out any unwanted influence of individual differences in task behaviour on the outcome of the data analysis.

The accuracy of detection was derived based on a combined measure encompassing the proportion of hits and correct rejections for images from a single target category (e.g., natural targets and built distractors). It was controlled for any confounding influence by participant age and gender through incorporating the interaction between these two variables within the regression equation. In addition, it was controlled for familiarity through including the *Rural Experience* variable, derived by dividing the number of years spent living in a village, hamlet, and/or farm by participant age, as predictor variable. Note that when using the *lme4* procedure, the effect of variables entered in the equation is conditional on (i.e. controlled for) those entered previously.

All data with associated response times smaller than 150 ms or greater than 1,000 ms were regarded as non-response data. A lower 150 ms response time threshold was chosen in agreement with the time interval required for target-specific neural activity to occur in higher-order visual areas (e.g., [41]). The upper 1,000 ms threshold was chosen in line with previous studies in which the Go/No-Go paradigm has been applied (e.g., [15]). Only response times associated with correct go responses were analyzed.

### Results

#### Accuracy

**Fig 2:**
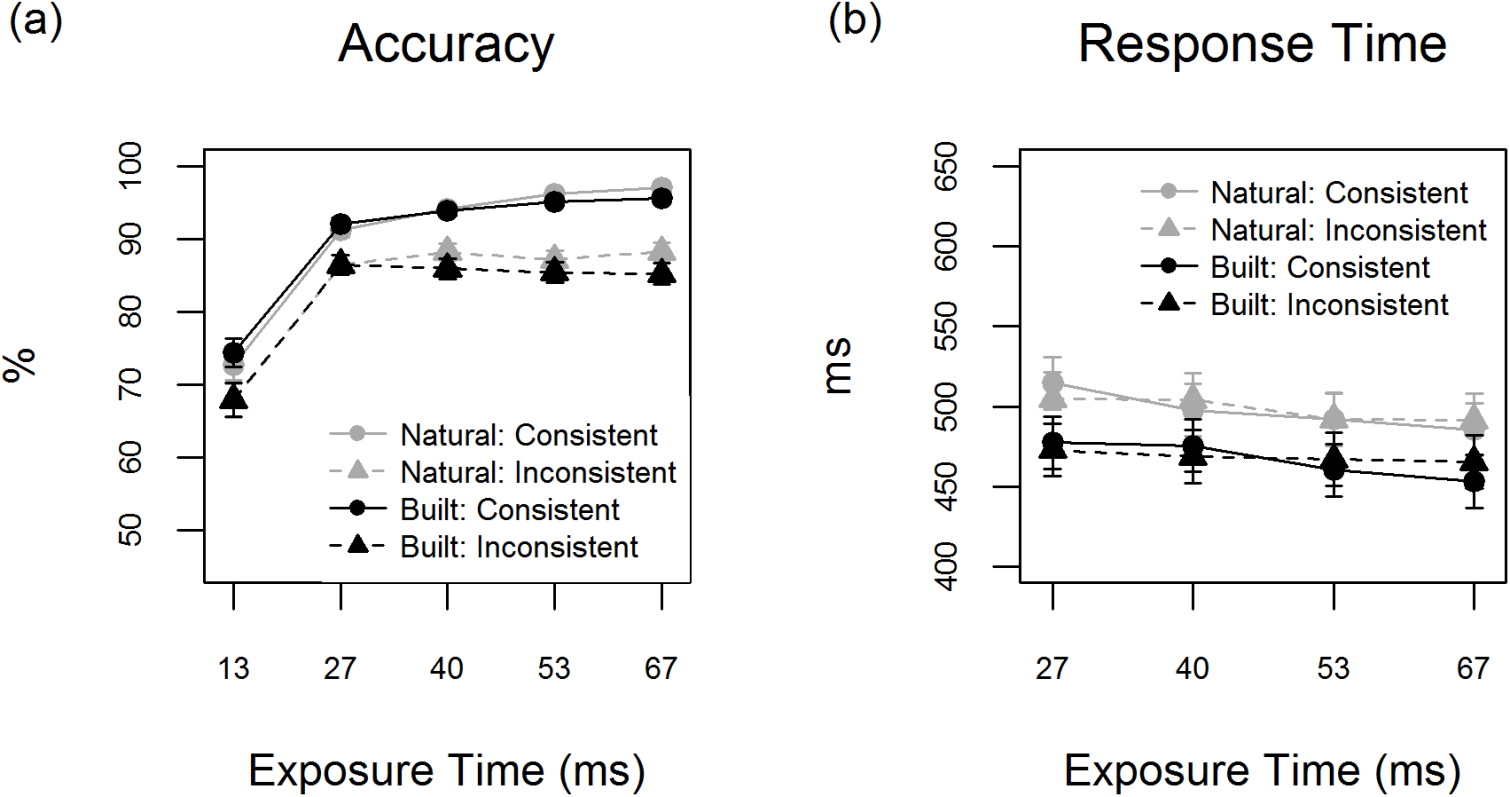
The results of Experiment 1 showing the (a) mean accuracy (%) and (b) mean response time (ms) associated with detection of image targets varying in scene category (natural or built) and consistency (consistent or inconsistent) for different exposure times. Error bars depict mean ± 1 SE.

To contrast the accuracy of natural and built target scene detection, separately for consistent and inconsistent image categories, a generalized linear mixed model (GLMM) was fitted with both participant number and image ID as random factors. In addition, it included as fixed factors a two-way interaction term between Gender and Age, Rural Experience and a three-way nested interaction term between ET (13, 27, 40, 53 or 67 ms), Target Category (natural or built) and Consistency (consistent or inconsistent). Concerning the effect of Target Category, higher accuracy of natural than built scene detection was observed with regard to both the *consistent* and the *inconsistent* image categories for the 67 ms ET (see Fig 2A & Table 1). In addition, *inconsistent natural* targets were detected more accurately than *inconsistent built* targets for the 40 ms ET. Finally, non-image control trials were more often misclassified as natural than as built targets (*M* = 18.6%, *SE* = 4.80 & *M* = 5.6%, *SE* = 1.75; *b* = 1.32, *SE* = .13, *z =* 10.2, *p* < .001).

**Table 1:** The effect of target category (natural or built) on accuracy and response time (RT) for different levels of image consistency and exposure time (ET) in Experiment 1. A negative test statistic denotes lower accuracy / shorter RTs for built than natural scenes.

		Accuracy				RT			
Consistency	ET	*b*	*SE*	*z*	*p*	*b*	*SE*	*t*	*p*
Consistent	13 ms	.09	.07	1.33	.184	x	x	x	x
Consistent	27 ms	.10	.10	1.06	>.250	-37.0	4.96	-7.46	< .001
Consistent	40 ms	-.05	.11	-.47	>.250	-22.0	4.85	-4.54	< .001
Consistent	53 ms	-.26	.13	-1.95	.051	-31.5	4.77	-6.61	< .001
Consistent	67 ms	-.42	.14	-2.91	.004	-32.4	4.74	-6.83	< .001
Inconsistent	13 ms	.00	.07	.04	>.250	x	x	x	x
Inconsistent	27 ms	.01	.09	.11	>.250	-31.8	5.78	-5.51	< .001
Inconsistent	40 ms	-.20	.10	-2.08	.037	-35.3	5.74	-6.15	< .001
Inconsistent	53 ms	-.15	.09	-1.56	.120	-25.1	5.72	-4.40	< .001
Inconsistent	67 ms	-.26	.10	-2.74	.006	-26.1	5.65	-4.62	< .001

Accuracy of detection was negatively influenced by inconsistent objects for both natural and built scene detection across all ETs (all *p*’s < .008, see S1 Table). The effect of consistency was similar across natural and built target categories as signified by an absence of significant Target Category x Consistency interactions (see S2 Table).

#### Response time

Next, all predictor and random variables from the GLMM were entered into a linear mixed model (LMM) for the analysis of response time (RT). It was chosen to withhold from analyzing the data associated with the 13 ms ET. This addressed a concern that a high proportion of correct responses for this ET may have resulted from guessing (accuracy associated with the 13 ms exposure time was still analysed in order to check the extent to which participants were guessing at the 13 ms exposure time). The RT analysis revealed that for the remaining ETs, the RTs associated with built scene detection were consistently shorter than those for natural scene detection (all *p*'s < .001). This finding applied to *consistent* and *inconsistent* scenes alike (see Fig 2B & Table 1).

Consistency condition did neither influence the speed of natural nor that of built scene detection (see S1 Table). The only exception was with regard to built scene detection for the 67 ms ET, which was slowed down for scenes including a natural element (*b* = 12.27, *SE* = 5.13, *t =* 2.39, *p* = .017). The analysis on RT did not reveal any significant interactions between Target Category and Consistency (see S2 Table).

### Discussion

In agreement with the hypothesis that built content is more salient than natural content, built scenes were detected with shorter response times than natural scenes. This applied to both the *consistent* and *inconsistent* images for each of the four exposure times in the 27–67 ms range. Although the accuracy of natural scene detection was also higher for some of the exposure times, this only applied to image presentations with longer durations. A speed-accuracy trade-off could therefore not account for the majority of the findings. Mask-only control trials were more often (mis)classified as natural rather than built scenes. This suggests that natural scenes are associated by participants with the absence of salient information.

A consistency effect could not be established with regard to the response time measure, except for built scene detection at the 67 ms exposure time. Also, it was comparable in size across natural and built scenes for each of the exposure times. This is in disagreement with the idea that built objects in natural scenes are more salient than natural objects in built scenes. The aim of the next experiment was to investigate if the lack of support for this hypothesis could be accounted for by colour speeding up natural scene detection disproportionally.

Colour information is extracted quickly and natural scenes are more colour diagnostic than built scenes [42]. Hence, colour might have been used as a heuristic for scene category.

## Experiment 2

Experiment 2 served to test the extent to which colour affects detection performance. To this end, we repeated Experiment 1 with an achromatic image set while retaining the original hypotheses. Images were controlled for colour, but not for any other image features that might be systematically associated with scene category (e.g., contrast, luminance, spatial frequency). The rationale for this is that these image properties fundamentally contribute to the structure of a scene (e.g., [11]) and we were primarily interested in gauging the saliency of the structural environment.

### Method

#### Participants and design

Forty students (30 female) with a mean age of 21.1 years (*SE* = .74) who had not participated in Experiment 1 were recruited from the University of Aberdeen. Participant requirements, reward, data-collection stoppage rule and experimental design were akin to Experiment 1.

#### Materials and procedure

All images used in Experiment 1 were converted into greyscale by applying a luminance conversion in Adobe Photoshop 12.0. This entailed that the red (R), green (G) and blue (B) channels describing each image pixel were converted into a single luminance value based on conventional use of a weighted average of the colour channels (.21 * R + .72 * G + .07 * B). This method was preferred over alternative solutions in order to preserve the contrast from the original full-colour image.

The experimental procedure matched that of Experiment 1 in all aspects.

### Results

#### Accuracy

**Fig 3:**
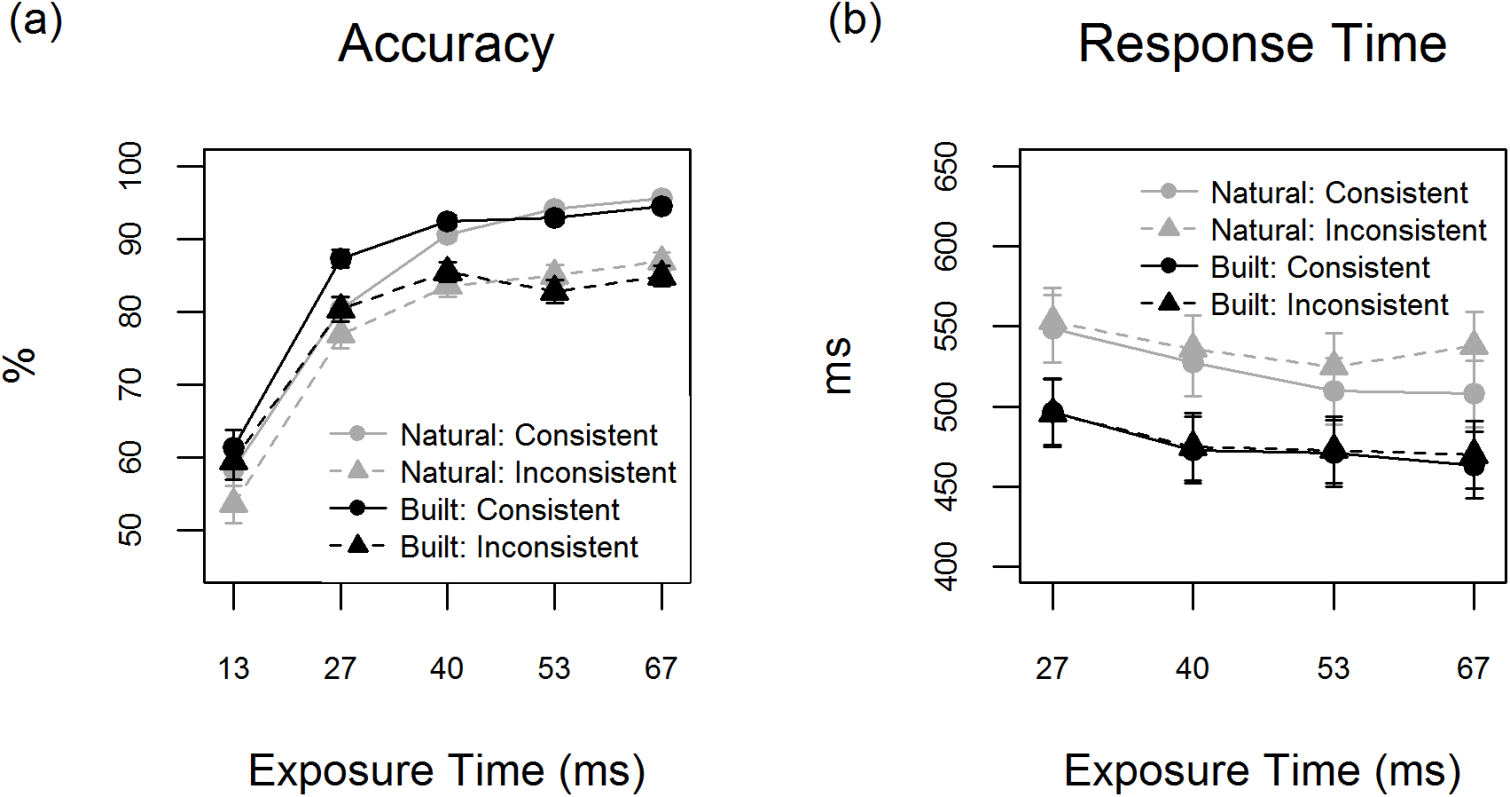
The results of Experiment 2 (achromatic scenes) showing the (a) mean accuracy (%) and (b) mean response time (ms) associated with detection of image targets varying in scene category (natural or built) and consistency (consistent or inconsistent) for different exposure times. Error bars depict mean ± 1 SE.

A GLMM identical to that used for Experiment 1 was fitted to analyze accuracy. This showed that the accuracy of *consistent built* target detection outweighed that of *consistent natural* targets for the 27 and 40 exposure times (ETs; see Fig 3A & Table 2). In addition, *inconsistent built* targets were detected with higher accuracy than *inconsistent natural* targets for the 13 and 27 ms ETs. Non-significant trends for higher accuracy of natural than built target detection could be observed with regard to both *consistent* and *inconsistent* scenes for the longer exposure times. Finally, mask-only control trials were more often misclassified as natural than as built scenes (*M* = 18.6%, *SE* = 4.80 & *M* = 5.6%, *SE* = 1.75; *b* = 1.42, *SE* = .13, *z* = 10.7, *p* < .001).

**Table 2:** The effect of target category (natural or built) on accuracy and response time (RT) for different levels of image consistency and exposure time (ET) in Experiment 2. A negative test statistic denotes lower accuracy / shorter RTs for built than natural scenes.

		Accuracy				RT			
Consistency	ET	*b*	*SE*	*z*	*p*	*b*	*SE*	*t*	*p*
Consistent	13 ms	.12	.06	1.95	.051	x	x	x	x
Consistent	27 ms	.51	.08	6.59	< .001	-51.9	5.71	-9.09	< .001
Consistent	40 ms	.23	.10	2.31	.021	-54.7	5.23	-10.5	< .001
Consistent	53 ms	-.22	.11	-1.96	.050	-38.8	5.12	-7.58	< .001
Consistent	67 ms	-.22	.12	-1.81	.071	-44.5	5.07	-8.78	< .001
Inconsistent	13 ms	.24	.07	3.38	< .001	x	x	x	x
Inconsistent	27 ms	.21	.08	2.54	.011	-57.2	6.75	-8.47	< .001
Inconsistent	40 ms	.14	.09	1.61	.107	-61.1	6.27	-9.74	< .001
Inconsistent	53 ms	-.16	.09	-1.82	.069	-51.9	6.23	-8.34	< .001
Inconsistent	67 ms	-.17	.09	-1.88	.060	-67.9	6.11	-11.1	< .001

The consistency effects previously established in Experiment 1 were largely replicated in the present experiment. This implies that consistency affected the accuracy of natural and built target scene detection for each of the ETs (all *p*’s < .014, see S3 Table). The only exception was the accuracy of built target detection for the 13 ms ET, which remained unaffected. Furthermore, a significant Target Category x Consistency interaction was established for the 27 ms ET, signalling a stronger consistency effect for built than for natural target detection (*b* = -.31, *SE* = .11, *z =* -2.74, *p =* .006). No Target Category x Consistency interactions were observed at any of the other exposure times (see S4 Table).

#### Response time

An LMM with identical variables to that in Experiment 2 was fitted in order to test the hypotheses related to the response time (RT) measure. Again, the data for the 13 ms ET were not analyzed as detection was close to chance-level. The results indicated that both *consistent* and *inconsistent built* scenes were detected with shorter RTs than their *natural* counterparts for each of the ETs (see Fig 3B & Table 2).

Scene consistency influenced RTs to natural scenes for the two longest ETs (*53 ms*: *b* = 15.25, *SE* = 5.88, *t =* 2.59, *p* = .010; *67 ms*: *b* = 30.0, *SE* = 5.79, *t =* 5.18, *p* < .001). There was no effect of consistency on built scene detection for any of the ETs (see S3 Table). The analysis of consistency further showed a significant Target Category x Consistency interaction for the 67 ms ET (*b* = -22.45, *SE* = 7.47, *t* = -3.01, *p* = .003), whilst not showing any significant effects for the other ETs (see S4 Table). This was due to a relatively profound detrimental influence of built elements on the speed of natural scene detection.

#### Discussion

The findings largely replicated those of Experiment 1. Both consistent and inconsistent built scene were detected with shorter response times than their natural counterparts at each of the exposure times, which supports the hypothesis that built scenes have more salient diagnostic features than natural scenes. None of these findings could be accounted for by a speed-accuracy trade-off as accuracy of detection was similar between natural and built targets at each of the exposure times. Again, mask-only control trials were more often (mis)classified as a natural than as a built scene.

In agreement with the hypothesis that built objects in natural scenes are more salient than the reverse, a stronger scene consistency effect on response time was observed for the natural than the built scene category at the 67 ms exposure time. Since this effect was not established when chromatic images were used in Experiment 1, it suggests that participants to some extent used colour as a heuristic for scene category. This is in agreement with previous studies showing that colour speeds up detection of colour-diagnostic scenes [42–43].

## Experiment 3

After having successfully demonstrated higher saliency of built than natural scene content, the aim of Experiment 3 was to test if saliency of scene content is indeed predictive of attention restoration. The procedure was based on a previous cognitive restoration study reported by Berman et al. [5, Experiment 2];. Participants performed the backward digit span (BDS) as a measure of directed-attention cognitive ability.

### Method

#### Participants and design

Forty-eight students (33 female) with a mean age of 21.4 years (*SE* = .13) were recruited from the University of Aberdeen. Participation was rewarded with course credit. Data collection ceased once the pre-determined sample size based on Berman et al. ([5]; Experiment 2) was reached.

The experiment used a 2 (Scene Category: Natural or Built) x 2 (Saliency: Salient or Non-Salient) between-subjects design.

#### Materials and procedure

The BDS-task was administered and scored as in the study of Berman et al. [5]. This entails that participants were presented with random digit sequences, which they were required to reproduce in the reverse order of presentation. The first digit sequence in the BDS-task was always three digits in length and after every second sequence one digit was added to the total. The final two sequences were nine digits in length, which entailed that a total of 14 digit sequences were presented. The BDS-score was derived by summing up the number of correctly reported sequences (irrespective of sequence length).

The Positive and Negative Affect Schedule (PANAS; [44]) was administered to gauge affect. Participants rated the extent to which 10 positive-affect and 10 negative-affect adjectives described their current mood, using a five-point Likert scale (1 = *slightly/not at all*, 5 = *extremely*).

**Fig 4:**
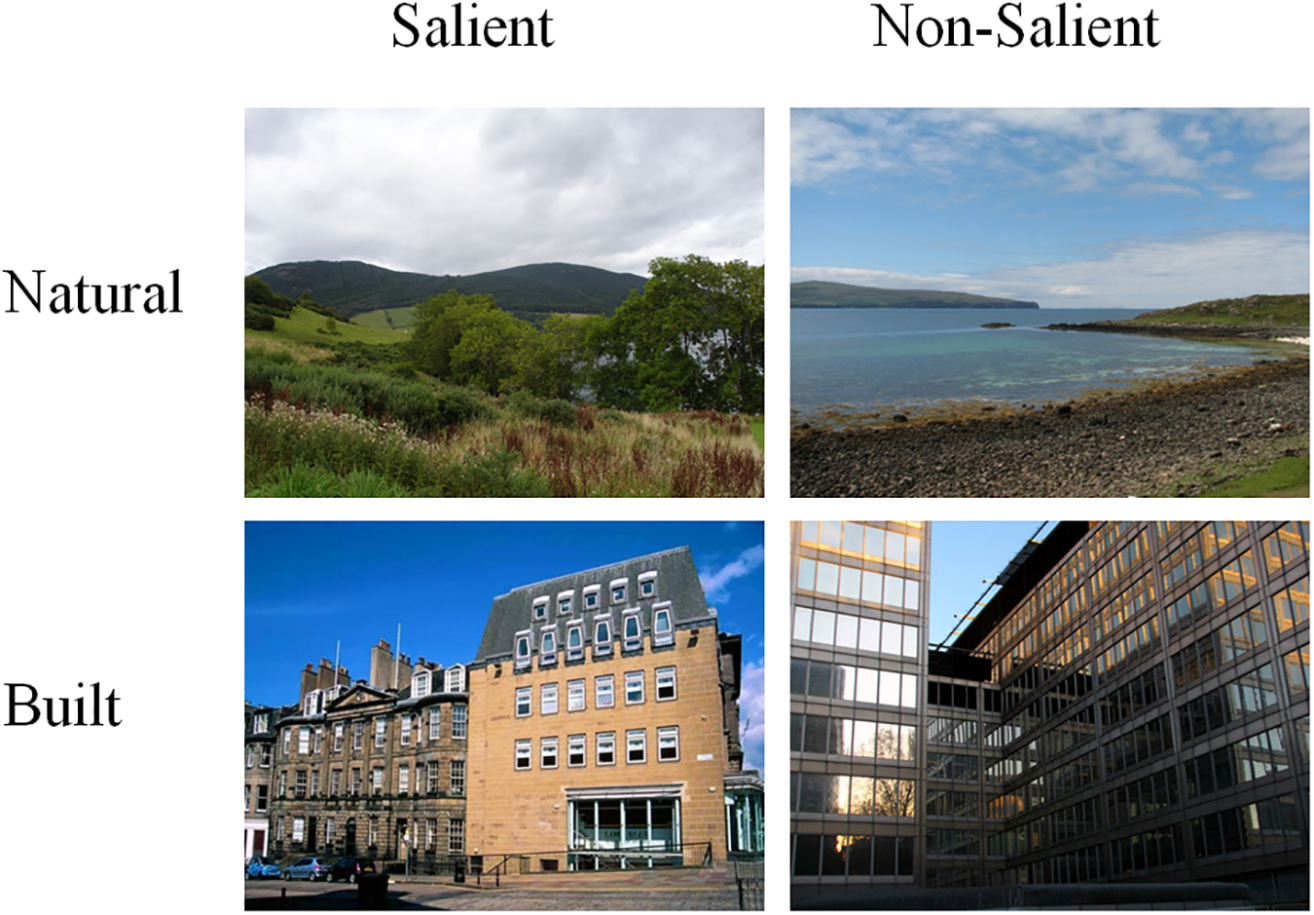
Examples of salient and non-salient natural and built images as used in Experiment 3.

Four sets of 50 highly variable natural and built images served as stimuli for the slideshow. All images were extracted from the set of 600 full-colour *consistent natural* and *built* images presented in Experiment 1. The images in both categories were selected based on aggregated mean response times associated with Correct Go responses in Experiment 1 and two pilot experiments described in Van der Jagt ([45], pp. 72–89). Those 50 natural and built scenes detected with the lowest and highest mean response times were classified as salient and non-salient images, respectively (see Fig 4). Image resolution, presentation apparatus and experimental software were identical to that employed in Experiments 1–2. The BDS and PANAS were administered as pencil-and-paper tasks.

Participants indicated their age and gender before completing the experimental tasks. They were randomly allocated to either one of the four image conditions. Images were presented for seven seconds each. Participants provided a preference rating on a 3-point Likert scale (1 = *low*, 3 = *high*) following image offset. Upon finishing the slideshow, the PANAS and BDS-task were completed a second time.

### Results

To investigate the effects of scene category and saliency on restoration, linear mixed models (LMMs) with participant number as random variable were fitted. These included as predictor variables the two-way interaction between Gender and Age and the three-way interaction between Scene Category (natural or built), Saliency (salient or non-salient) and Measure (pre- or post-slideshow). These showed no significant three-way interactions for BDS (*b* = .92, *SE* = .97, *t* = .95, *p* >.250) and the two affect variables (*positive affect*: *b* = -3.08, *SE* = 3.49, *t* = -.88, *p* >.250; *negative affect*: *b* = .17, *SE* = 1.88, *t* = .09, *p* >.250).

**Fig 5:**
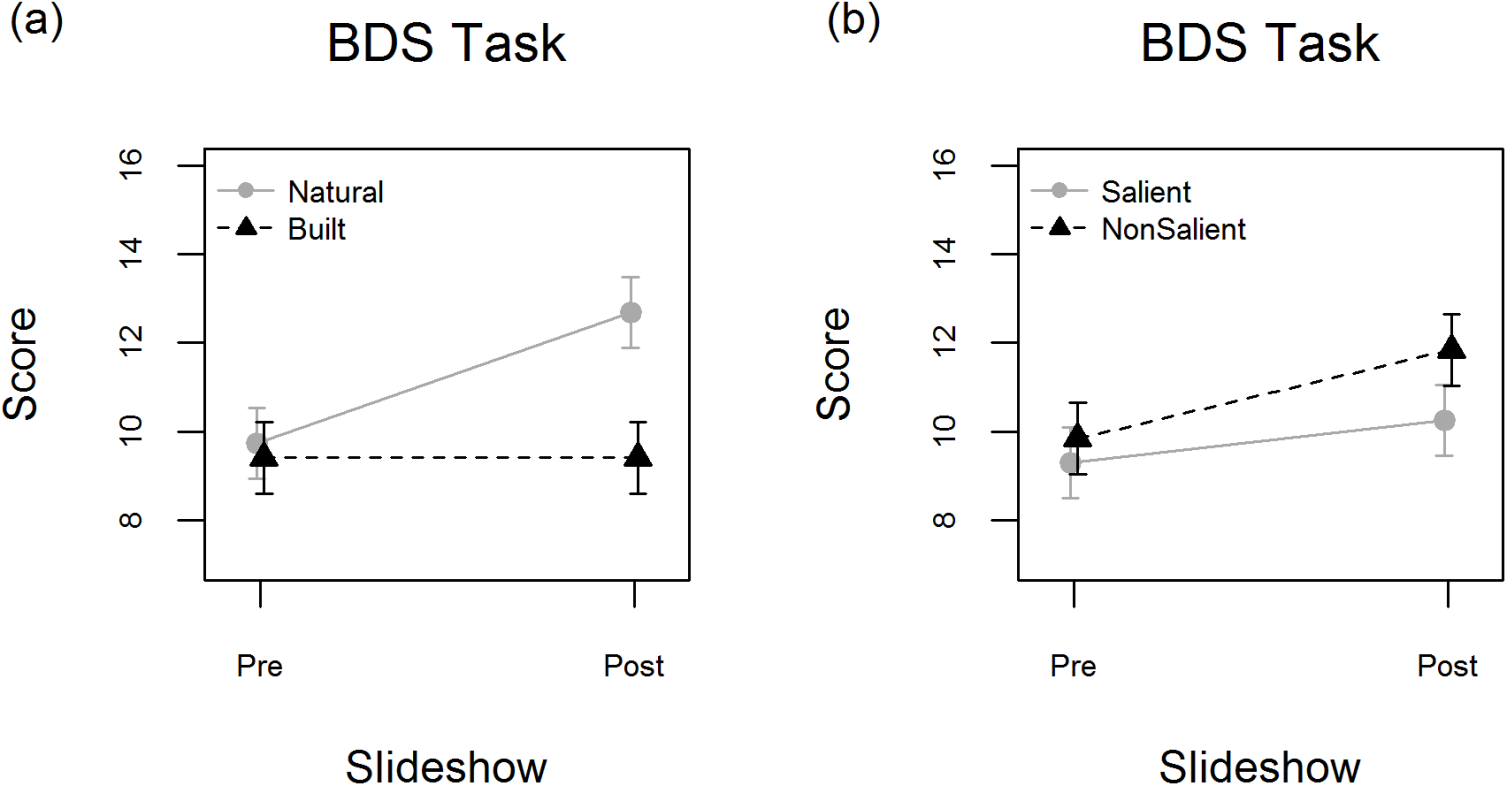
Pre- and post-slideshow Backward Digit Span (BDS) scores for (a) the Scene Category x Measure interaction and (b) the Saliency x Measure interaction. Error bars depict mean ± 1 SE.

Subsequently, LMMs in which the three-way interaction term was replaced by two two-way interaction terms (Scene Category x Measure & Saliency x Measure) were fitted. In agreement with the hypothesis, the LMM on BDS-score showed a significant Scene Category x Measure interaction (*b* = -2.96, *SE* = .48, *t* = -6.12, *p* < .001), which was due to natural scenes increasing digit span more strongly than built scenes (see Fig 5A). The Saliency x Measure interaction was also significant (*b* = 1.04, *SE* = .48, *t* = 2.16, *p* = .036). Fig 5B shows that this is due to salient scenes increasing digit span to a lesser degree than non-salient scenes. Positive and negative affect were not influenced by scene category (*positive affect*: *b* = -1.71, *SE* = 1.74, *t* = -.98, *p* >.250; *negative affect*: *b* = 1.17, *SE* = .93, *t* = 1.26, *p* = .22) or saliency (*positive affect*: *b* = -.79, *SE* = 1.74, *t* = -.45, *p* >.250; *negative affect*: *b* = -.83, *SE* = .93, *t* = -.90, *p* >.250) of images. The LMM on preference ratings (excluding the Measure predictor variable) showed a main effect of Scene Category, indicating that participants liked natural scenes better than built scenes (*b* = -.34, *SE* = .07, *t* = -5.03, *p* < .001). Preferences were not significantly different between salient and non-salient scenes (*b* = -.09, *SE* = .07, *t* = -1.38, *p* = .176).

### Discussion

Using an established restoration paradigm in combination with a new image set, we replicated the finding that natural scene content is cognitively more restorative than built scene content previous demonstrated by Berman et al [5]. Crucially, it was also shown that saliency, as it naturally varies with different scene content, significantly interacts with restoration effects: scenes lacking salient content were cognitively more restorative than scenes including salient content. The effect of saliency on the degree of cognitive restoration can be considered separately from the effect of natural-built scene category as an equal number of natural and built scenes were included in the groupings with high and low saliency. This finding provides empirical support for the assumption in ART that scenes which invoke soft fascination (i.e. that lack salient content) are more restorative than those triggering hard fascination (i.e. that have salient content). The effect of saliency on restoration is relatively weak when compared to the effect of scene category, which could potentially be explained by saliency having less of a role in explaining response times to natural scenes; instead diagnostic colour may have been a much more important variable. In this sense, the effect of saliency on restoration perhaps may not be considered fully independent of scene category.

## General discussion

The present study investigated the assumption in ART (e.g., [9]) that scenes which invoke soft fascination are more restorative than those triggering hard fascination. Specifically, we tested whether the saliency of scene content can function as an empirically-derived indicator of hard and soft fascination. Our results show that built scene content is more salient than natural content (Experiments 1 & 2) and provide some indication that, when controlled for colour, built objects within a natural scene are more salient than natural objects in built environments (Experiment 2). Moreover, Experiment 3 was the first study in the literature to demonstrate that both the saliency level of image content, as well as its (natural or built) scene category, influence cognitive restoration.

The disruptive effect of a semantically inconsistent element on accuracy of natural and built scene detection is in agreement with research showing an inhibitory relationship between object- and scene-centered neural pathways [46] and studies showing a negative influence of semantically inconsistent objects on accuracy of scene detection [47, 16]. Additional studies are required to explore the extent to which inconsistent objects (e.g., wind turbines in a landscape) associated with saliency are predictive of restoration and to better understand what features tend to influence the typically high saliency of built environments. Candidate features could include straight edges and lines (in particular two-dimensional edge content such as corners or occlusions), which have been shown to be predictive of scene fixations using eye tracking [48–49]. Indeed, a recent study showed a lack of straight lines and low hue diversity to be predictive of participants' ratings of naturalness [50]. For that reason, it is not unlikely that there is a relationship between saliency and perceived naturalness in both natural and built scenes. Future work could also look into the question of the extent to which saliency plays a role in predicting cognitive restoration in response to natural scenes, as it may be that people do not rely on the perception of salient low-level features when detecting natural scenes, instead using global information or simply responding to a *lack* of salient features.

Another interesting research avenue to explore would be the question of whether all salient features, not just those diagnostic of natural or built scene content, predict cognitive restoration to a similar extent. To this end, computational modelling could be applied to create image sets that vary in saliency of scene content based on a particular type of low-level feature contrast (e.g., luminance or colour) predicting saliency, but not on others. These image sets could then be compared on their potential to evoke cognitive restoration using a behavioural experiment similar to that in Experiment 3.

Another interesting question to explore is whether, given the speed of saliency-based attention of <50 ms [11], participants really need longer image exposures (7 s p/image in the present study) for restoration from mental fatigue to occur. Restorative nature experiences following brief interactions with nature have been anecdotally described [51–52], but to our knowledge temporal aspects of restoration have not yet been studied systematically. On what grounds would we predict exposures of several seconds or more per image are required for cognitive restoration? According to ART the purported link between saliency and attention restoration is that executive attention is required to inhibit attentional capture from irrelevant stimuli in order to maintain a task focus [5, 10]. However, there was no clear need for participants to inhibit irrelevant stimuli in the present study. That is, participants were not asked to perform any cognitive tasks concurrently with observing the slideshow and no incentive was provided for inhibiting the materials displayed on the screen. An alternative explanation may be offered by the biased competition theory [53]. According to this theory, stimuli compete for visual representation in the ventral visual pathway and this competition is biased to items with either top-down or bottom-up saliency. Beck and Kastner [54] provide an excellent overview of research in support of this claim. For instance, it has been shown for neurons in the ventral visual stream—a cortical network important in driving bottom-up attention [55–56]—that their activation in response to a particular reference stimulus displayed in their receptive field is suppressed when an additional probe stimulus is presented, indicating mutually suppressive interactions rather than independent processing [57]. Furthermore, they also demonstrated that the level of suppression is enhanced when the saliency of the probe stimulus is increased. Interestingly, single-cell recording studies have shown that executive attention can be used to overcome a suppressive influence by a stimulus. Kastner and Ungerleider ([58], p. 323) write about this: "[…] attention may resolve the competition among multiple stimuli by counteracting the suppressive influences of nearby stimuli, thereby enhancing information processing at the attended location. This may be an important mechanism by which attention filters out unwanted information from cluttered visual scenes".

It follows from this that executive attention is required when visually exploring scenes to suppress involuntary attention to salient elements, and deployment of executive attention in this process is likely a function of the saliency of scene content. A tentative theory could therefore be that exposure to scenes with salient content depletes the executive attention resource because executive attention is required in eliminating the suppression of non-salient scene content. If this or a similar theory predicting a positive relationship between saliency and executive attention required for detailed scene processing is valid, we would indeed expect to only find a relationship between saliency of scene content and cognitive restoration with longer exposure times, allowing for a number of fixations on a scene.

This study directly supports ART by showing that visual information invoking soft fascination (i.e. non-salient scene content) is cognitively restorative. Objectively measuring scene saliency may therefore provide a rigorous empirical means to establish the restorative potential of different types of environments, both natural and built. We argue that these findings can contribute to a more nuanced understanding of what entails a ‘restorative’ environment that moves beyond the current approach of treating natural and built scene categories as homogenous entities [59].

## Supporting information

S1 Supporting Information (DOCX)

S1 Table The effect of consistency (consistent or inconsistent) on accuracy and response time (RT) for different levels of target category (TC) and exposure time (ET) in Experiment 1. (DOCX)

S2 Table The effect of the consistency x target category interaction on accuracy and response time (RT) for different levels of exposure time (ET) in Experiment 1. (DOCX)

S3 Table The effect of consistency (consistent or inconsistent) on accuracy and response time (RT) for different levels of target category (TC) and exposure time (ET) in Experiment 2. (DOCX)

S4 Table The effect of the consistency x target category interaction on accuracy and response time (RT) for different levels of exposure time (ET) in Experiment 2. (DOCX)

S1 Fig A plot depicting the frequency at which images within the final databases for the four image categories were rated at each of the possible combined content scores. (TIF)
