# Supplementary material for: A view not to be missed: Salient scene content interferes with cognitive restoration
Source: PLoS One. 2017 Jul 19;12(7):e0169997. doi: 10.1371/journal.pone.0169997 (PMC5516974; doi:10.1371/journal.pone.0169997)
Supplement: S1 Supporting Information — (DOCX) [file pone.0169997.s001.docx]

**Supporting Information**

**Methodological Considerations**

In previous natural/built Go/No-Go studies (e.g., Joubert et al., 2007), image sets have typically been derived from commercial image libraries incorporating professional photographs organized alongside content categories. For the purpose of the present study, however, a different approach was taken. Firstly, rather than relying on commercial image libraries, we opted for the extraction of images from online repositories with non-commercial images. That is, commercial stock libraries typically depict a limited range of rather beautiful and spectacular scene content. Consequently, these image databases do not provide a representative cross-section of natural and built environments. In addition, care was taken to filter out any images in which the photographer created an illusionary scene representation by applying a shallow depth-of-field or photo montage techniques, which might influence the ease of scene detection. Finally, images depicting humans were deliberately excluded from the image database. This decision was motivated on the idea that the perception of human figures might serve as a proxy for the built environment in which they are likely to have a higher likelihood of being present.

The present study also diverged from previous research regarding the procedure for allocating images to the natural and built scene categories respectively. Previously, image allocation has typically been based on pre-existing categorizations within the commercial image repository (e.g., beaches 🡪 natural; streets 🡪 built). However, this practice was deemed unsuitable for the present research since there is unlikely to be a clear boundary line between human conceptualisations of the natural and the built scene categories. For instance, many scenes which do not show man-made objects nonetheless have clear signs of human influence (e.g., farmland). Scenes which cannot be straightforwardly allocated to either the natural or built scene category should be avoided because semantic ambiguity is likely to slow down response times or interfere with response accuracy in a detection task. In order to do so, the scene category of images was pre-validated for the purpose of the present study. A large pool of images, pre-categorized as either natural or built, was run through a participant panel who rated each image on natural and built content. Subsequently, scenes which could be regarded as boundary cases because of similar ratings on both the natural and built scales were filtered out. In addition, scenes could only be classified as inconsistent when (a) pre-classified as inconsistent by the experimenter and (b) when participants reported a degree of both natural and built content.

**Image validation procedure**

One hundred participants (71 female) participated in the image validation study. The ages of the participants varied from 18 to 51 years with a mean age of 20.6 years old (*SE* = 0.53). All participants were undergraduate students from the University of Aberdeen with normal or corrected-to-normal vision and were rewarded by course credit for participation. Participants rated the degree to which images matched the "natural" and "built" scene category labels (presented along with a number of other variables) using a Likert scale ranging from 1 (*strongly disagree*) to 7 (*strongly agree*)^[[1]](#footnote-1)^. Each participant individually completed the task on a computer situated in a PC lab. Each participant completed 80 trials; a group of 20 participants was required for each of the 1600 images (400 consistent natural, 400 consistent built, 400 inconsistent natural, 400 inconsistent built) to be rated on all items once. Completing the questionnaire took approximately 75 minutes.

Overall, Krippendorff’s Alpha coefficients indicated reasonable inter-rater agreement regarding the degree of natural and built content of the images within the database (*natural: α* = 0.73, 95% CI [0.70, 0.76]; *built: α* = 0.71, 95% CI [0.68, 0.74]), which justified the use of the natural and built content measures for the purpose of image validation. In the first step of image validation, the natural and built content ratings, provided by each observer, were combined into a single combined content variable by subtracting the built from the natural content ratings. This combined content measure could vary between the extremes of -6 and 6, with a score of -6 indicating a scene rated with the maximum built content score and the minimum natural content score and a score of 6 indicating a scene rated with the maximum natural content score and the minimum built content score. Furthermore, scenes with scores of 0 represented a perfect boundary case, with an equivalent “degree” of natural and built content. The combined measure served as the basis upon which decisions regarding the inclusion of images within the database were taken.

In the subsequent stage of image validation, the combined content measures from each of the five judges were pooled together for each image. For an image to be classified as natural it needed on average to be judged more natural than built, hence have a positive score on the combined content measure. Conversely, for an image to be incorporated within the built scene category, a negative mean rating on the combined content measure was required. For inconsistent images to be selected, an additional requirement was that the element of inconsistency was perceived as such by participants. The measure of scene inconsistency was derived using individual natural and built content ratings. For a natural scene to be classified as inconsistent it had to be both pre-classified as *inconsistent natural* and on average not have received both high natural and low built content ratings (mean of combined content measure ≥ 5). Similarly, for a built scene to be classified as inconsistent it had to be pre-classified as such and not have received high built and low natural content ratings (mean of combined content measure ≤ -5). In addition, consistent and inconsistent images for which inter-rater agreement was particularly low were excluded based on a standard deviation criterion (*SD* ≥ 3). The criterion was set in such way as to exclude those scenes for which disagreement was particularly high, while still being sufficiently lenient to allow for an image database sufficiently large for usage in a detection experiment.

Applying these criteria limited the natural consistent image database to 371 scenes, whereas the built consistent database was brought down to 350 scenes. To minimize overlap with the inconsistent scenes, the databases for the natural and built consistent image categories were each further brought down to 300 images by removing the 71 natural images with lowest mean score and the 50 built images with highest mean score on the combined content measure. For the inconsistent scenes, applying the criteria for selection resulted in 230 natural and 251 built scenes. For reasons of research design, the total inconsistent image set was then further brought down to 460 images (50% natural) by random exclusion of 21 built scenes. The inter-rater agreement for the validated image sets was high with respect to both the natural and built content measures (*natural: α* = 0.82, 95% CI [0.80, 0.83]; *built: α* = 0.81, 95% CI [0.79, 0.83]). This showed that the validation procedure had been successful in filtering out those scenes for which observers showed clear disagreement in terms of content ratings.

In addition, a plot depicting the frequency at which images from the four categories were scored on each of the levels of the combined content scale, following the filtering out of boundary case images, revealed relatively little overlap between the distributions for each of the four image categories (see S1 Figure). The majority of consistent natural and built scenes scored at the high and low extremes of the scale respectively, indicating images from both categories were relatively prototypical. Furthermore, none of the participants rated a consistent scene within the validated image database as a scene more representative of the “opposite” scene category (e.g., a natural scene rated more strongly as built than natural). With regard to the group of inconsistent scenes selected following validation, it was observed that the frequency at which these are rated at the extremes of the scale is much lower than for consistent scenes. Although inconsistent scenes were at times rated as better representatives of the “opposite” scene category, such occurrences were relatively rare. This signalled that, although the inconsistent element clearly affected ratings on the combined content measure, images of this type were relatively easy to categorize along the natural-built spectrum.

1. An initial pilot study showed stronger inter-rater agreement when participants were asked to rate scenes in terms of ‘natural’ and ‘built’ content than when other labels, such as ‘pristine’, ‘organic’ (for the natural scene category) or ‘man-made’, ‘urban’ or ‘artificial’ (for the built scene category), were used. Therefore, ‘natural’ and ‘built’ were used as scene category labels both within the image validation pilot study and the Go/No-Go experiments in the present study. In previous Go/No-Go studies ‘man-made’ has typically been used instead of ‘built’ as scene category label. [↑](#footnote-ref-1)
