# Supplementary material for: A view not to be missed: Salient scene content interferes with cognitive restoration
Source: PLoS One. 2017 Jul 19;12(7):e0169997. doi: 10.1371/journal.pone.0169997 (PMC5516974; doi:10.1371/journal.pone.0169997)
Supplement: S1 Table — (DOCX) [file pone.0169997.s002.docx]

Table S1.
*The Effect of Consistency (Consistent or Inconsistent) on Accuracy and Response Time (RT) for Different Levels of Target Category (TC) and Exposure Time (ET) in Experiment 1.*

|  |  |  | Accuracy | | | |  | RT | | | |
| --- | --- | --- | --- | --- | --- | --- | --- | --- | --- | --- | --- |
| TC | ET |  | *b* | *SE* | *z* | *p* |  | *b* | *SE* | *t* | *p* |
| Natural | 13 ms |  | -.22 | .08 | -2.64 | .008 |  | x | x | x | x |
| Natural | 27 ms |  | -.48 | .11 | -4.56 | < .001 |  | -9.82 | 5.41 | -1.82 | .070 |
| Natural | 40 ms |  | -.74 | .12 | -6.40 | < .001 |  | 6.57 | 5.36 | 1.23 | .220 |
| Natural | 53 ms |  | -1.30 | .13 | -10.2 | < .001 |  | .26 | 5.37 | .05 | >.250 |
| Natural | 67 ms |  | -1.46 | .14 | -10.6 | < .001 |  | 5.95 | 5.30 | 1.12 | >.250 |
| Built | 13 ms |  | -.30 | .08 | -3.61 | < .001 |  | x | x | x | x |
| Built | 27 ms |  | -.58 | .11 | -5.38 | < .001 |  | -4.68 | 5.35 | -.88 | >.250 |
| Built | 40 ms |  | -.91 | .11 | -8.01 | < .001 |  | -6.76 | 5.26 | -1.29 | .199 |
| Built | 53 ms |  | -1.19 | .12 | -10.0 | < .001 |  | 6.65 | 5.16 | 1.29 | .197 |
| Built | 67 ms |  | -1.30 | .12 | -10.7 | < .001 |  | 12.27 | 5.13 | 2.39 | .017 |
