# Supplementary material for: A view not to be missed: Salient scene content interferes with cognitive restoration
Source: PLoS One. 2017 Jul 19;12(7):e0169997. doi: 10.1371/journal.pone.0169997 (PMC5516974; doi:10.1371/journal.pone.0169997)
Supplement: S2 Table — (DOCX) [file pone.0169997.s003.docx]

Table S2.
*The effect of the Consistency x Target Category interaction on Accuracy and Response Time (RT) for Different Levels of Exposure Time (ET) in Experiment 1.*

|  |  | Accuracy | | | |  | RT | | | |
| --- | --- | --- | --- | --- | --- | --- | --- | --- | --- | --- |
| ET |  | *b* | *SE* | *z* | *p* |  | *b* | *SE* | *t* | *p* |
| 13 ms |  | -.09 | .10 | -.88 | >.250 |  | x | x | x | x |
| 27 ms |  | -.09 | .14 | -.69 | >.250 |  | 5.02 | 7.84 | .64 | >.250 |
| 40 ms |  | -.16 | .15 | -1.10 | >.250 |  | -13.4 | 7.60 | -1.77 | .077 |
| 53 ms |  | .10 | .16 | .65 | >.250 |  | .44 | 7.54 | .85 | >.250 |
| 67 ms |  | .17 | .17 | .98 | >.250 |  | 6.86 | 6.76 | 1.02 | >.250 |
