# Supplementary material for: A view not to be missed: Salient scene content interferes with cognitive restoration
Source: PLoS One. 2017 Jul 19;12(7):e0169997. doi: 10.1371/journal.pone.0169997 (PMC5516974; doi:10.1371/journal.pone.0169997)
Supplement: S3 Table — (DOCX) [file pone.0169997.s004.docx]

Table S3

*The Effect of Consistency (Consistent or Inconsistent) on Accuracy and Response Time (RT) for Different Levels of Target Category (TC) and Exposure Time (ET) in Experiment 2.*

|  |  |  | Accuracy | | | |  | RT | | | |
| --- | --- | --- | --- | --- | --- | --- | --- | --- | --- | --- | --- |
| TC | ET |  | *b* | *SE* | *z* | *p* |  | *b* | *SE* | *t* | *p* |
| Natural | 13 ms |  | -.20 | .08 | -2.60 | .009 |  | x | x | x | x |
| Natural | 27 ms |  | -.21 | .09 | -2.46 | .014 |  | 4.49 | 6.47 | .69 | >.250 |
| Natural | 40 ms |  | -.65 | .10 | -6.49 | < .001 |  | 8.57 | 6.00 | 1.43 | .153 |
| Natural | 53 ms |  | -1.05 | .11 | -9.43 | < .001 |  | 15.25 | 5.88 | 2.59 | .010 |
| Natural | 67 ms |  | -1.16 | .12 | -9.62 | < .001 |  | 30.00 | 5.79 | 5.18 | < .001 |
| Built | 13 ms |  | x | x | x | x |  | x | x | x | x |
| Built | 27 ms |  | -.52 | .09 | -5.61 | < .001 |  | -0.74 | 6.01 | -.12 | >.250 |
| Built | 40 ms |  | -.74 | .11 | -6.98 | < .001 |  | 2.17 | 5.53 | .39 | >.250 |
| Built | 53 ms |  | -1.0 | .10 | -9.51 | < .001 |  | 2.13 | 5.51 | .39 | >.250 |
| Built | 67 ms |  | -1.12 | .11 | -9.93 | < .001 |  | 6.53 | 5.43 | 1.20 | .229 |
