# Supplementary material for: A view not to be missed: Salient scene content interferes with cognitive restoration
Source: PLoS One. 2017 Jul 19;12(7):e0169997. doi: 10.1371/journal.pone.0169997 (PMC5516974; doi:10.1371/journal.pone.0169997)
Supplement: S4 Table — (DOCX) [file pone.0169997.s005.docx]

Table S4.
*The effect of the Consistency x Target Category interaction on Accuracy and Response Time (RT) for Different Levels of Exposure Time (ET) in Experiment 2.*

|  |  | Accuracy | | | |  | RT | | | |
| --- | --- | --- | --- | --- | --- | --- | --- | --- | --- | --- |
| ET |  | *b* | *SE* | *z* | *p* |  | *b* | *SE* | *t* | *p* |
| 13 ms |  | -.11 | 0.09 | 1.15 | >.250 |  | x | x | x | x |
| 27 ms |  | -.31 | .11 | -2.74 | .006 |  | -6.34 | 9.27 | -.68 | >.250 |
| 40 ms |  | -.09 | .13 | -.66 | >.250 |  | -5.79 | 8.28 | -.70 | >.250 |
| 53 ms |  | 0.05 | .14 | .35 | >.250 |  | -13.14 | 7.94 | -1.65 | .098 |
| 67 ms |  | 0.05 | .15 | .35 | >.250 |  | -22.45 | 7.47 | -3.01 | .003 |
