# Supplementary figures and images for: A view not to be missed: Salient scene content interferes with cognitive restoration
Source: PLoS One. 2017 Jul 19;12(7):e0169997. doi: 10.1371/journal.pone.0169997 (PMC5516974; doi:10.1371/journal.pone.0169997)

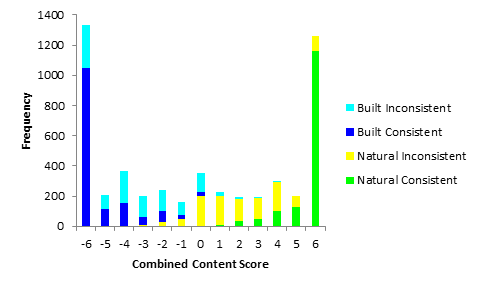

Supplement: S1 Fig — (TIF) [file pone.0169997.s006.tif]
